# Viability of primary osteoblasts after treatment with tenofovir alafenamide: Lack of cytotoxicity at clinically relevant drug concentrations

**DOI:** 10.1371/journal.pone.0169948

**Published:** 2017-02-09

**Authors:** Christian Callebaut, Yang Liu, Darius Babusis, Adrian Ray, Michael Miller, Kathryn Kitrinos

**Affiliations:** 1 Clinical Virology Department, Gilead Sciences, Foster City, California, United States of America; 2 Drug Metabolism Department, Gilead Sciences, Foster City, California, United States of America; Asociacion Civil Impacta Salud y Educacion, PERU

## Abstract

Tenofovir alafenamide (TAF) is a phosphonoamidate prodrug of the nucleotide HIV reverse transcriptase inhibitor tenofovir (TFV). TAF is approved for the treatment of HIV-1 infection as part of the single-tablet regimen containing elvitegravir, cobicistat, emtricitabine, and TAF. When dosed once-daily, TAF results in approximately 90% lower levels of plasma TFV and a 4-fold increase in intracellular TFV-diphosphate (TFV-DP) in PBMCs compared with the TFV prodrug tenofovir disoproxil fumarate (TDF). Several antiretrovirals, including TDF, have been associated with bone mineral density decreases in patients; the effect of clinically relevant TAF concentrations on primary osteoblast viability was therefore assessed in vitro. Studies in PBMCs determined that a 2-hour TAF exposure at concentrations similar to human plasma C_max_ achieved intracellular TFV-DP levels comparable to those observed after the maximum recommended human dose of 25 mg TAF. Comparable intracellular TFV-DP levels were achieved in primary osteoblasts with 2-hour TAF exposure daily for 3 days at concentrations similar to those used for PBMCs (100–400 nM). No change in cell viability was observed in either primary osteoblasts or PBMCs. The mean TAF CC_50_ in primary osteoblasts after 3 days of daily 2-hour pulses was >500 μM, which is >1033 times higher than the TAF maximum recommended human dose plasma C_max_. In summary, primary osteoblasts were not preferentially loaded by TAF compared with PBMCs, with comparable TFV-DP levels achieved in both cell types. Furthermore, there was no impact on osteoblast cell viability at clinically relevant TAF concentrations.

## Introduction

The development of antiretroviral drugs for HIV-1 infected patients has dramatically improved quality of life, with the average life expectancy increasing approximately 10 to 15 years since the introduction of highly active antiretroviral therapy [1, 2]. As a consequence, additional health issues may arise due to long term treatment with antiretroviral drugs [3–6]. One long-term side effect that has been associated with multiple HIV-1 treatments and HIV infection itself, is a decrease in bone mineral density (BMD), which can lead to osteopenia and osteoporosis [7–12].

Tenofovir disoproxil fumarate (TDF) is a prodrug of the nucleotide reverse transcriptase inhibitor tenofovir (TFV), which was approved for the treatment of HIV-1 in 2001 [13] and chronic hepatitis B in 2008. TDF was developed to improve upon the low TFV permeability and allow for its systemic delivery [14]; however, TDF is quickly metabolized to TFV [15]. Inefficient TDF delivery to peripheral cells results in high systemic concentrations of TFV after dosing in order to achieve sufficient levels of tenofovir diphosphate (TFV-DP) in target cells [16]. Tenofovir alafenamide (TAF) is a new prodrug of TFV, developed to load target cells more efficiently while lowering TFV systemic levels [17, 18], and consequently reduce off-target TFV exposure [19–21]. In clinical studies evaluating TAF-containing regimens in HIV or HBV infected patients, TAF is more stable in human plasma and delivers TFV into lymphoid cells more efficiently than TDF [17]. 4 fold higher TFV-DP levels were achieved in PBMCs as well as approximately 90% lower TFV plasma concentrations compared with TDF-containing regimens; notably, an improved renal and bone safety profile was observed for TAF groups as compared to TDF groups [22, 23].

Tenofovir-related nephrotoxicity has been infrequently observed in clinical trials with TDF-containing regimens; although rare, acute renal failures have been reported in patients [24]. Disruption of normal kidney function is known to perturb renal phosphate handling [25]. As it has been established that bone growth is linked to phosphate regulation, the impact of high TFV systemic exposure on bone mass density (BMD) may be mediated by disruption of phosphate regulation in the kidney [25].

In clinical studies with TAF-containing regimens, the substantial decrease in plasma TFV observed compared with TDF-containing regimens likely contributes to the difference in BMD changes observed [22, 23]. However, the increased levels of TFV-DP in PBMCs suggest that TAF may be more efficiently distributed to other cells in the body compared with TDF. The goal of this study was to characterize the effect of clinically relevant concentrations of TAF on primary osteoblasts, the bone forming cells.

## Materials and methods

### Reagents and cells

TAF, TFV, and nelfinavir (NFV) were synthesized by Gilead Sciences (Foster City, CA). Lopinavir (LPV) was purchased from Toronto Research Chemicals (North York, ON, Canada). Nyosil-M25 oil was purchased from Nye Lubricants (Fairhaven, MA). PBMCs were obtained from the Stanford Blood Bank (Palo Alto, CA) and maintained in RPMI-1640 culture medium acquired from Invitrogen (Carlsbad, CA) containing 15% fetal bovine serum, 100 units/mL of penicillin, 100 μg/mL of streptomycin, and 20 IU/mL of interleukin-2 (IL-2) produced by Sigma (St. Louis, MO) at a density of 2.5 × 10^6^ cells/mL. Human proliferating osteoblast cells (lot #6122) were obtained from Lonza (Walkersville, MD) in 96-well or 6-well plates and maintained in Lonza’s osteoblast basal medium, supplemented with 10% fetal bovine serum, ascorbic acid and gentamicin/amphotericin-B.

### PBMC loading assay

PBMCs were activated in RPMI-1640 culture medium with 1 μg/mL phytohemagglutinin (Sigma) for 3 days. After 3 days, cells were maintained in RPMI-1640 culture medium without phytohemagglutinin for 1 week followed by seeding in 12-well plates at a density of 5 × 10^6^ cells per well in 2.5 mL of medium containing TAF at concentrations of 0, 0.0137, 0.0412, 0.124, 0.370, 1.111, 3.333, or 10 μM. Two TAF dosing conditions were evaluated, a continuous incubation and a 2-hour exposure with subsequent washout. For the continuous TAF incubation, PBMCs were treated for 6, 24, or 48 hours. For the 2-hour pulse, PBMCs were treated with TAF for 2 hours, washed twice with fresh medium, and then reseeded in new 12-well plates without TAF treatment for 4, 22, or 46 hours. At the indicated time points, two aliquots of 2 × 10^6^ cells for each TAF concentration were transferred to two 1.5 mL microfuge tubes containing 0.5 mL Nyosil-M25 oil for immediate cell extraction (see below).

### Primary osteoblast loading assay

Human proliferating osteoblast cells seeded in 6-well plates were immediately incubated at 37°C and 5% CO_2_ upon arrival. The following day, the osteoblast culture medium was replaced with 2.5 mL of fresh medium, taking care to not disrupt the osteoblasts, and then incubated at 37°C and 5% CO_2_ for 3 additional days until the osteoblasts reached 80% confluence. The osteoblast culture medium was then replaced with 2.5 mL fresh medium containing 0, 0.124, 0.370, 1.111, 3.333, or 10 μM TAF. Two TAF dosing conditions were evaluated, a 2-hour exposure with immediate harvest and a 2-hour exposure followed by a 22-hour washout. At each time point, two aliquots of 5 × 10^5^ cells for each TAF concentration were transferred to two 1.5 mL microfuge tubes containing 0.5 mL Nyosil-M25 oil for immediate cell extraction (see below). Additional osteoblast loading assays were conducted for 3 days with daily 2-hour TAF exposures and 22-hour washouts. The cell handling prior to drug treatment was identical to what was done in the single-pulse assays described above. The TAF concentrations evaluated in the 3-day assay were 0, 0.1, 0.2, and 0.4 μM. Osteoblasts were harvested at 2 and 22 hours after the third TAF pulse. Two aliquots of 5 × 10^5^ cells for each TAF concentration were transferred to two 1.5 microfuge tubes containing 0.5 mL Nyosil-M25 oil for immediate cell extraction (see below). These experiments were repeated four times.

### Cell extraction

Harvested cells were spun through the Nyosil-M25 oil layer at 13,000 rpm for 45 seconds. The microfuge tubes were then washed twice with phosphate-buffered saline to remove any extracellular TFV-DP, being careful not to disrupt the oil layer. After washing, the Nyosil-M25 oil layer was removed from the tubes, without disrupting the cell pellet. Intracellular TFV-DP was then extracted from the cell pellet by resuspending cells in 0.5 mL 70% methanol at −80°C for at least 30 minutes. The resulting supernatant was transferred to a new microfuge tube for measurement of intracellular TFV-DP (see below).

### Measurement of intracellular nucleotide analog metabolism

Using methods previously described [26], methanol extracts were dried in a centrifuging evaporator (Genevac, Stone Ridge, NY) and samples were reconstituted with an aqueous solution of ammonium phosphate (1 mM, pH 7) containing an internal standard (500 nM 2-chloro-adenosine triphosphate; Cl-ATP, Sigma Aldrich, St. Louis, MO). Analytes were separated using a 50 × 2 mm x 2.5 μ Luna C18(2) HST column (Phenomenex, Torrance, CA) connected to a LC-20ADXR (Shimadzu, Columbia, MD) ternary pump system and HTS PAL autosampler (LEAP Technologies, Carrboro, NC). A multistage linear gradient from 10% to 50% acetonitrile in a mobile phase containing 3 mM ammonium formate (pH 5.0) with 10 mM dimethylhexylamine at a flow rate of 150 μL/min was used to separate analytes. Detection was performed on an API4000 (Applied Biosystems, Foster City, CA) MS/MS operating in positive ion and multiple reaction monitoring modes. TAF metabolites TFV (parent mass-to-charge ratio (*m/*z) → monitored daughter ion; 288 → 176.1), tenofovir monophosphate (TFV-MP; 368 → 176.1) and TFV-DP (448 → 176.1) were quantified using an eight-point standard curve (with Cl-ATP as internal standard [542.2 → 170.2]), ranging in concentration from 0.572 to 1,250 pmol/million cells in osteoblasts and from 0.091 to 200 pmol/million cells in PBMCs, and were prepared in cell extract from respective untreated cells. Reported concentrations (in μM) were calculated based on an estimated intracellular volume of either 4.2 pL/cell (osteoblasts) or 0.2 pL/cell (PBMCs).

### Cell viability assay

Human proliferating osteoblast cells seeded in 96-well plates were immediately incubated at 37°C and 5% CO_2_ upon arrival. The following day, the osteoblast culture medium was replaced with 100 μL of fresh medium, taking care not to disrupt the cells, and then incubated at 37°C and 5% CO_2_ for 3 additional days until the osteoblasts reached approximately 80% confluence. The osteoblast culture medium was then replaced with 100 μL fresh medium containing 0, 2.0, 3.9, 7.8, 15.6, 31.3, 62.5, 125, 250, or 500 μM TAF, with triplicate wells for each TAF concentration. Osteoblasts were treated for 3 days with daily 2-hour exposures and 22-hour washouts. Cell viability was measured 22 hours after the third TAF pulse. TFV and two HIV protease inhibitors, NFV and LPV, were used as controls. Osteoblasts were continuously incubated with controls for 3 days. The TFV concentrations evaluated were 0, 0.15, 0.45, 1.4, 4.1, 12.3, 37, 111, 333, and 1,000 μM. The NFV concentrations evaluated were 0, 6.3, 12.5, 25, and 50 μM and the LPV concentrations evaluated were 0, 12.5, 25, 50, and 100 μM.

As previously described [27], osteoblast cell viability was assessed following drug treatment by adding Cell-Titer Glo reagent (Promega, Madison, WI) to the treated cells and measuring luminescence on a VICTOR^™^ luminescence plate reader (Perkin-Elmer, Waltham, MA). Each luminescence signal was divided by the signal of the untreated cells to determine a relative percent signal for each drug tested and 50% cytotoxic concentration values (CC_50_) were calculated using GraphPad Prism software (GraphPad Software, La Jolla, CA).

## Results

### TAF loading in PBMCs

TAF loading studies were conducted in PBMCs to determine the in vitro concentration(s) of TAF that resulted in intracellular TFV-DP levels comparable to those observed in PBMCs in vivo with 25 mg TAF in a Phase 1 clinical study [28]. PBMC pharmacokinetic results from this 10-day monotherapy study demonstrated that subjects achieved a mean TFV-DP concentration of 0.677 μM predose on day 10, with a 2-fold increase to 1.493 μM 12 hours postdose (Table 1). A broad range of TAF concentrations (0.01–10 μM) was evaluated to ensure the target TFV-DP concentration was achieved.

**Table 1:** Intracellular levels of TFV-DP in PBMCs for subjects receiving 25 mg TAF.

PK Parameter^a^	Mean Intracellular TFV-DP	
	ng/10^6^ cells	μM^b^
Pre-dose	0.0605	0.677
12 Hours Postdose	0.1335	1.493

^a^As observed in TAF monotherapy study (data from study GS-US-120-0104)

^b^Calculated from TFV-DP concentration in ng/10^6^ cells using a PBMC intracellular volume of 0.2 pL/cell

Two TAF dosing conditions were evaluated in the PBMC loading assays, a continuous incubation and a single 2-hour exposure with washout. Continuous incubation was evaluated since this is a standard method for cell loading assays. However, the TAF PK parameters measured in the Phase 1 clinical study demonstrate that TAF is rapidly absorbed (median T_max_ approximately 0.50 hours) and has a short plasma half-life (t_1/2_ approximately 0.40 hours) [28]. Therefore, in order to better mimic in vivo TAF exposure conditions, a single 2-hour exposure with washout was also evaluated.

Overall, TFV-DP levels were dose proportional for both dosing conditions. The target TFV-DP concentration of 0.677–1.493 μM (Table 1) was achieved after 6 hours of continuous dosing with 0.0137–0.124 μM TAF, the lowest doses evaluated (data not shown). For the single 2-hour pulse, the target TFV-DP concentration was achieved with 0.370 μM TAF by 6 hours (4 hours postpulse) (Table 2), which is in alignment with the mean TAF plasma C_max_ of 0.484 μM observed at steady state for patients receiving 25 mg TAF [28].

**Table 2:** Intracellular TFV-DP levels detected in PBMC with a single 2-hour TAF pulse and washout.

TAF (μM)^a^	TFV-DP Concentration (μM) ^b^ After a 2 Hour Pulse		
	6 hours	24 hours	48 hours
0.0137	BLQ	BLQ	BLQ
0.0412	BLQ	BLQ	BLQ
0.124	BLQ	BLQ	BLQ
0.370	1.89±1.05	1.35±0.13	0.95±0.17
1.111	7.05±0.21	4.01±0.31	3.17±0.91
3.333	19.9±1.7	13.5±1.2	7.03±0.53
10.000	73.5 ^c^	46.7±1.7	24.5±1.8

^a^ Data generated from at least 2 independent experiments

^b^ Mean ± standard deviation; BLQ = below limit of quantitation (limit of quantitation: 0.5 μM)

^c^ Single value available for this treatment condition

### TAF loading in primary osteoblasts

TAF loading studies using a single 2-hour exposure and washout were conducted in primary osteoblasts using similar TAF concentrations to those evaluated in PBMCs (0.124–10 μM). Similar to what was observed in PBMCs, TFV-DP levels were dose proportional across all TAF concentrations at the 2-hour time point and for the 0.370–3.333 μM doses at the 24-hour time point (Table 3). The target TFV-DP concentration was achieved with 0.370–1.111 μM TAF 2 hours postpulse, similar to what was observed in PBMCs by 6 hours (4 hours postpulse).

**Table 3:** Intracellular TFV-DP levels detected in primary osteoblasts with a single 2-hour TAF pulse and washout.

TAF (μM) ^a^	TFV-DP Concentration (μM)^b^ After a2 Hour Pulse	
	2 hours	24 hours
0.124	0.298±0.013	0.305±0.201
0.370	0.812±0.079	0.469±0.180
1.111	1.917±0.347	1.390±0.054
3.333	5.429±0.825	4.024±1.397
10.000	18.786±1.414	9.690±5.775

^a^ Data generated from at least 2 independent experiments

^b^ Mean ± standard deviation; Calculated from an osteoblast intracellular volume of 4.2 pL/cell

Additional studies were performed in primary osteoblasts to determine if multiple TAF doses had any impact on TFV-DP levels. Three days of daily 2-hour TAF pulses were conducted, with primary osteoblasts harvested at 2 and 24 hours after the final dose (50 and 72 hours).

TAF concentrations of 0.100 μM, 0.200 μM, and 0.400 μM were selected based on the results of the single-pulse experiments in both PBMCs and primary osteoblasts (Tables 2 and 3).

The TFV-DP levels achieved in primary osteoblasts at all TAF concentrations after 3 days of 2-hour pulses were similar to what was observed after a single 2-hour pulse, suggesting no accumulation of TFV-DP in primary osteoblasts (Table 4). In addition, the TFV-DP levels achieved were similar to those observed with a single pulse of TAF in PBMCs (Table 2). Primary osteoblasts pulsed three times with 0.400 μM TAF achieved a TFV-DP concentration of 0.914 μM (Table 4), while PBMCs pulsed once with 0.370 μM TAF achieved a TFV-DP concentration of 1.35 μM (Table 2).

**Table 4:** Intracellular TFV-DP levels detected in primary osteoblasts with 3 days of 2-hour TAF pulses and washout.

TAF (μM) ^a^	TFV-DP (μM)^b^	
	50 hours (2h after 3^rd^ TAF pulse)	72 hours (24h after 3^rd^ TAF pulse)
0.100	0.271±0.016	0.145±0.026
0.200	0.790±0.284	0.395±0.063
0.400	1.511±0.465	0.914±0.071

^a^ Data generated from 4 independent experiments

^b^ Mean ± standard deviation; Calculated using an osteoblast intracellular volume of 4.2 pL/cell.

### Evaluation of cell viability

The primary osteoblast assay was also used to evaluate the potential cytotoxicity of TAF using 3 days of daily 2-hour pulses. The concentration range of TAF evaluated ranged from clinically relevant plasma concentrations (Table 1) to supratherapeutic TAF concentrations. Two HIV protease inhibitors (PIs) nelfinavir (NFV) and lopinavir (LPV) were selected as positive cytotoxic controls and evaluated after 3 days of continuous incubation (consistent with clinical exposure) [29, 30]. Protease inhibitors, including LPV, have been shown to be associated with BMD loss in vivo [7]. NFV has also been shown to effect primary osteoblast growth in vitro [31].

When TAF was evaluated using the daily 2-hour pulse, no cytotoxicity was observed; therefore, the mean CC_50_ after 3 days of treatment was >500 μM (the highest concentration evaluated), which is >1033 times higher than the mean TAF plasma C_max_ of 0.484 μM (Table 5) [28, 32]. The estimated TAF in vitro selectivity ratio (in vitro CC_50_/ in vitro TAF exposure) for osteoblast toxicity is >2500 (>500 μM/ 0.200 μM).

**Table 5:** Clinical C_max_ and observed CC_50_ values for compounds evaluated in primary osteoblasts.

Clinical Data		Osteoblast In Vitro Assay Data				Ratio
Drug	C_max_ (μM)	Drug	Treatment	n	CC_50_ (μM)^a^	CC_50_/ C_max_
TAF 25 mg QD	0.484 (TAF)	TAF	2 hour pulse	5	>500	>1033
TDF 300 mg QD	1 (TFV)	TFV	Continuous	4	>1000	>1000
NFV 1250 mg BID	7 (NFV)	NFV	Continuous	4	23.5 ± 4.5	3.4
LPV 800 mg QD^b^	18.7 (LPV)	NFV	Continuous	4	33.5 ± 3.8	1.8

^a^Mean ± standard deviation

^b^Boosted with 200 mg ritonavir

The mean CC_50_ of TFV was >1,000 μM, which is >1,000 times higher than the mean TFV plasma C_max_ of 1 μM for 300 mg TDF and >20,000 higher than the mean TFV plasma C_max_ of 0.05 μM for 25 mg TAF (Table 5) [28, 33]. For the HIV-1 PIs, the mean CC_50_ of NFV and LPV were 23.5 μM and 33.5 μM, respectively. Both CC_50_ values were relatively close to their mean plasma C_max_, 3.4 and 1.8 times higher than the reported mean plasma C_max_ for NFV and LPV, respectively. However, as NFV and LPV are highly bound to serum protein (90–95%), the selectivity values are estimated to be approximately 10–20 times higher after adjustment for protein binding.

## Discussion

Although TDF is well tolerated and has been a preferred backbone in HIV therapy, it has been associated with effects on renal function and BMD [24]. A large multiyear observation study conducted with the Veterans Affairs Clinical Case Registry found an association between cumulative TDF exposure and an increased rate of fractures, suggesting that assessment of bone toxicity may be warranted before and after TDF-containing antiretroviral therapy initiation [34].

Results from this study demonstrate that osteoblasts do not represent a cell type that is more sensitive to TAF, as primary osteoblasts and PBMCs treated with TAF concentrations consistent with therapeutic exposure achieved comparable TFV-DP levels. Additionally, in cells exposed to 3 days of 2-hour daily TAF pulses, there was no accumulation of TAF compared with a single 2-hour TAF pulse. The similar levels between PBMCs and osteoblasts suggest that TAF conversion to active moiety of TFV-DP is consistent between the two cells types. Previous studies have documented that in PBMCs, TAF is predominantly converted to TFV by cathespin A [35]. While we have not identified which enzyme(s) contribute to the conversion of TAF to TFV in osteoblasts, this study suggests that the kinetics of TAF conversion to TFV in PBMCs and osteoblasts is similar.

At clinically relevant concentrations of TAF, there was no cytotoxicity observed on primary osteoblasts. Because TAF is rapidly absorbed and has a short plasma half-life [28], comparisons to clinical C_max_ using a 2-hour exposure and washout method is physiologically relevant. No effect on cell viability was seen with TAF at concentrations up to 500 μM. Results from another study found that treatment of primary mouse osteoblasts for 3 days with 50 μM TDF or higher significantly reduced cell viability [27]. However, the primary mouse osteoblasts were continuously exposed to TDF for 3 days, which is a less clinically relevant exposure compared with the daily TAF 2-hour pulse method used in this study.

The CC_50_ values of the HIV-1 PIs nelfinavir and lopinavir were only 3.4- and 1.8-fold higher than their respective clinical C_max_ values (not taking into account 90% plasma protein binding seen for most PIs). Cellular toxicity in cell lines for these PIs have been documented in vitro [36] and have been associated with BMD decreases in vivo [7]. The CC_50_ value for TAF was >500 μM, which is more than 1,033-fold above the mean plasma C_max_; this value was achieved using a 2-hour exposure and washout indicating that TAF is not cytotoxic at clinical exposures. Using a less biologically relevant continuous incubation method, the TAF CC_50_ value on osteoblasts was 10.4 μM, which is consistent with CC_50_ values observed on several other cell lines and cell types treated by continuous incubation, including PBMCs [18]. These results suggest that osteoblasts do not represent a more sensitive cell type for TAF.

Another finding from this study is that comparable levels of TFV-DP are achieved in PBMCs and primary osteoblasts with similar in vitro TAF exposure. Results from a dog tissue distribution study using [^14^C]-labeled TAF found that TAF-related radioactivity in bone was significantly lower compared with PBMCs [17]. While the dog study did not evaluate levels of TAF in the different bone cell types, this result may suggest that in vivo primary osteoblast exposure to TAF may be lower than PBMC exposure, potentially further increasing the TAF safety margin.

Bone homeostasis is controlled by the balance between the osteoblast and osteoclast activities, allowing continuous modeling of the bone tissue. One limitation of this study is that osteoblasts were studied independently of osteoclasts. Some studies have shown that activation of osteoclasts occurs in HIV+ subjects [37], suggesting that osteoclasts may play a role in the reduced BMD in infected subjects. Additionally, this study focuses on cell viability, which may not have captured all potential aspects of osteoblast damage by the drugs used in these studies.

Our methodology does not allow us to investigate the established link between kidney function in phosphate metabolism and bone formation [25]. While the molecular mechanism by which TDF is associated with decreased BMD has not been definitively identified, clinical studies have shown reduced effects on markers of renal function and changes in BMD for E/C/F/TAF relative to E/C/F/TDF [22, 23, 38, 39], including the clinical data obtained with E/C/F/TAF in renally impaired patients [40]. Combined, the lack of effect on osteoblasts observed in this study and the findings from clinical studies may suggest that the mechanism for changes in BMD in patients taking TDF containing regimens is secondary to the effects of higher circulating levels of TFV and, resulting, higher exposure to the kidney.

In conclusion, primary osteoblasts were not preferentially loaded by TAF relative to PBMCs, with comparable TFV-DP levels achieved in both cell types. Furthermore, there was no toxicity on osteoblast in vitro at therapeutic or supratherapeutic TAF concentrations.
